# Short-course quinazoline drug treatments are effective in the *Litomosoides sigmodontis* and *Brugia pahangi* jird models

**DOI:** 10.1016/j.ijpddr.2019.12.001

**Published:** 2019-12-10

**Authors:** Marc P. Hübner, Emma Gunderson, Ian Vogel, Christina A. Bulman, K.C. Lim, Marianne Koschel, Alexandra Ehrens, Stefan J. Frohberger, Martina Fendler, Nancy Tricoche, Denis Voronin, Andrew Steven, Victor Chi, Malina A. Bakowski, Ashley K. Woods, H. Michael Petrassi, Case W. McNamara, Brenda Beerntsen, Laura Chappell, William Sullivan, Mark J. Taylor, Joseph D. Turner, Achim Hoerauf, Sara Lustigman, Judy A. Sakanari

**Affiliations:** a Institute for Medical Microbiology, Immunology and Parasitology, University Hospital Bonn, Germany; b Dept. of Pharmaceutical Chemistry, University of California San Francisco, San Francisco, CA, USA; c Laboratory of Molecular Parasitology, Lindsley F. Kimball Research Institute, New York Blood Center, New York, USA; d Centre for Drugs and Diagnostics, Dept. of Tropical Disease Biology, Liverpool School of Tropical Medicine, Liverpool, UK; e Calibr, a Division of The Scripps Research Institute, La Jolla, CA, USA; f Veterinary Pathobiology, University of Missouri-Columbia, Columbia, MO, USA; g Dept. of Molecular, Cell and Developmental Biology, University of California, Santa Cruz, CA, USA

**Keywords:** Quinazoline, *Litomosoides sigmodontis*, *Brugia pahangi*, Filaria, Doxycycline, *Wolbachia*, Macrofilaricidal, Microfilariae

## Abstract

The quinazolines CBR417 and CBR490 were previously shown to be potent anti-wolbachials that deplete *Wolbachia* endosymbionts of filarial nematodes and present promising pre-clinical candidates for human filarial diseases such as onchocerciasis. In the present study we tested both candidates in two models of chronic filarial infection, namely the *Litomosoides sigmodontis* and *Brugia pahangi* jird model and assessed their long-term effect on *Wolbachia* depletion, microfilariae counts and filarial embryogenesis 16−18 weeks after treatment initiation (wpt). Once per day (QD) oral treatment with CBR417 (50 mg/kg) for 4 days or twice per day (BID) with CBR490 (25 mg/kg) for 7 days during patent *L. sigmodontis* infection reduced the *Wolbachia* load by >99% and completely cleared peripheral microfilaremia from 10–14 wpt. Similarly, 7 days of QD treatments (40 mg/kg) with CBR417 or CBR490 cleared >99% of *Wolbachia* from *B. pahangi* and reduced peritoneal microfilariae counts by 93% in the case of CBR417 treatment. Transmission electron microscopy analysis indicated intensive damage to the *B. pahangi* ovaries following CBR417 treatment and in accordance filarial embryogenesis was inhibited in both models after CBR417 or CBR490 treatment. Suboptimal treatment regimens of CBR417 or CBR490 did not lead to a maintained reduction of the microfilariae and *Wolbachia* load. In conclusion, CBR417 or CBR490 are pre-clinical candidates for filarial diseases, which achieve long-term clearance of *Wolbachia* endosymbionts of filarial nematodes, inhibit filarial embryogenesis and clear microfilaremia with treatments as short as 7 days.

## Highlights

• CBR417 and CBR490 provide long-term effects in 2 chronic filaria jird models.
• CBR417 and CBR490 deplete >99% *Wolbachia* in *B. pahangi* and *L. sigmodontis* filariae.
• CBR417 and CBR490 clear *L. sigmodontis* microfilariae after 10–14 weeks.
• CBR417 and CBR490 inhibit filarial embryogenesis in both models.
• Suboptimal doses do not maintain reduction of microfilariae and *Wolbachia*.

## Graphical abstract

**Figure:**
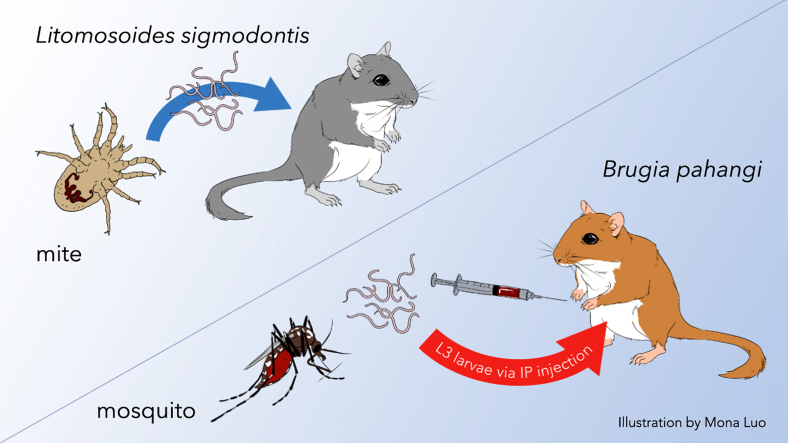

## 1 Introduction

The neglected tropical disease known as onchocerciasis or river blindness is caused by the filarial nematode *Onchocerca volvulus.* The disease is characterized by adult worms residing in subcutaneous nodules and the release of microfilariae (mf) into the skin by the female worms (Hoerauf et al., 2011). Disease pathology occurs as a result of mf death in dermal and ocular tissues leading to release of somatic inflammatory molecules including release of the filarial bacterial endosymbiont, *Wolbachia.* Host immunopathology induced by liberated antigens leads to onchodermatitis, keratitis and eventual visual impairment (Saint Andre et al., 2002; Gillette-Ferguson et al., 2004; Turner et al., 2009; Tamarozzi et al., 2011). Since the 1970s several control programs, initially based on vector control, were conducted to prevent the transmission of onchocerciasis but later included mass drug administration (MDA) with ivermectin (IVM) (Hoerauf et al., 2011). The recently stated sustainable development goal by the United Nations is to eliminate the transmission of infection and disease, and thus eliminate onchocerciasis by 2030 (WHO, 2019). All current control strategies for the control and elimination of onchocerciasis rely on MDA of IVM, which temporarily inhibits embryogenesis within female adult worms and thereby decreases transmission of mf. However, IVM has no macrofilaricidal efficacy, i.e. does not kill the adult worms, and therefore must be given annually or bi-annually over the reproductive lifespan of the adult worms, which can be up to 15 years for *O. volvulus*. More recently, moxidectin has been implemented as a novel treatment option for onchocerciasis. Moxidectin also targets the mf stage but leads to a longer suppression of the microfilaridermia and is therefore expected to reduce the MDA rounds required (Opoku et al., 2018). However, administration of the microfilaricides IVM and moxidectin by MDA is an issue in areas co-endemic for loiasis, since *Loa loa* infections with high microfilaremia (>30,000 mf/mL) can lead to life-threatening serious adverse events (SAEs) upon IVM administration (Gardon et al., 1997; Taylor et al., 2010; Opoku et al., 2018). Thus, drugs that can be safely administered in areas co-endemic for *O. volvulus* and *L. loa*, i.e. those that have macrofilaricidal but no or little microfilaricidal activity are critically needed to support the sustainable development goal of eliminating onchocerciasis within the next 10 years.

*O. volvulus*, like most human-pathogenic filariae, harbors intracellular endosymbiotic bacteria, *Wolbachia*, which are important for the fecundity and viability of the filariae (Hoerauf et al., 1999, 2000). Targeting *Wolbachia* with 4–6 weeks of doxycycline therapy leads to permanent sterilization of the adult female filariae and provides macrofilaricidal efficacy over time (~2 years) (Hoerauf et al., 2001). Since doxycycline was shown to be the first safe macrofilaricidal drug available and *L. loa* lacks *Wolbachia* endosymbionts (Büttner et al., 2003; Grobusch et al., 2003; McGarry et al., 2003; Desjardins et al., 2013), a major effort was undertaken to identify anti-*Wolbachia* drug candidates with an increased potency and shorter treatment regimens (Bakowski and McNamara, 2019). Quinazolines represent such a novel class of drug candidates with a potent anti-*Wolbachia* efficacy. Recently, two quinazolines, CBR417 and CBR490, were identified and optimized through a primary cell-based high-content imaging screen and an *ex vivo* worm-based validation assay (Bakowski et al., 2019). Both compounds showed excellent anti-*Wolbachia* potency *in vitro* and *ex vivo* and mediated rapid clearance of *Wolbachia* in *in vivo* rodent models of filariasis (Bakowski et al., 2019). A structurally related azaquinazoline candidate, AWZ1066S, has entered formal preclinical development (Hong et al., 2019).

In this study, we further characterized the long-term efficacy of CBR417 and CBR490 in jirds chronically infected with the filarial nematodes *Litomosoides sigmodontis* and *Brugia pahangi.* Our study demonstrates that mf initially decreased in numbers 8–10 weeks following treatment, but they rebounded when animals were given sub-optimal doses. However, higher optimal doses of the two anti-*Wolbachia* compounds were efficacious in inhibiting embryogenesis, decreasing the number of mf and decreasing *Wolbachia* titers in adult female worms in both animal models. Our studies therefore provide supporting evidence to nominate these quinazoline compounds as preclinical drug candidates for treatment of human filarial diseases.

## 2 Methods

### 2.1 *Litomosoides sigmodontis in vivo* studies

#### 2.1.1 Animal infections

Female jirds (*Meriones unguiculatus*) 6- to 8-weeks of age were obtained from Janvier Labs (Saint-Berthevin, France) and housed in individually ventilated cages at the animal facility of the Institute for Medical Microbiology, Immunology and Parasitology, University Hospital Bonn, in accordance with the European Union's animal welfare guidelines. All protocols were approved by the Landesamt für Natur, Umwelt und Verbraucherschutz, Cologne, Germany (AZ 84–02.04.2015.A507). For *L. sigmodontis* infection, jirds were exposed to *Ornithonyssus bacoti* mites infected with infective third-stage larvae (L3). All animals from one experiment were infected with the same batch of mites to ensure equal infection rates. Only mf positive jirds were included in the study and treatment started 13–16 weeks post-infection (wpi). As jirds are highly susceptible for infection with *L. sigmodontis* and harbor the infection for more than 1 year, differences in the treatment start date by a few weeks do not affect the outcome of the study (Morris et al., 2013).

#### 2.1.2 Drug formulations and dosages

Only mf positive jirds were included in the studies and received oral gavages with 2.5 mL/kg of the formulated drugs. Doxycycline (Sigma-Aldrich) was formulated in distilled water, and CBR417 as well as CBR490 were provided by Calibr at Scripps Research and formulated in 40% cyclodextrine diluted in distilled water.

#### 2.1.3 *L. sigmodontis* jird study I

Five experimental groups were tested in this study and treatment was initiated 13 wpi (Table 1). Group 1 remained untreated and was used as control (n = 6). Group 2 received bi-daily (BID) 40 mg/kg doxycycline for 14 consecutive days (n = 6). Group 3 received 50 mg/kg CBR417 once-per-day (QD) for 4 consecutive days (n = 6). Group 4 received 75 mg/kg CBR490 BID for 7 consecutive days (n = 6) and group 5 received 25 mg/kg CBR490 BID for 7 consecutive days (n = 6). Necropsies were performed at 18 weeks after the start of treatment (weeks post-treatment, wpt). At necropsy *L. sigmodontis* adult worms were isolated from the thoracic cavity and enumerated as previously described (Hübner et al., 2019a).

**Table 1:** Dosing regimen for *L. sigmodontis*-infected jirds in study I.

Group	Treatment	n =	Dose (mg/kg)	Daily doses	Duration (d)
1	Untreated	6	–	–	–
2	Doxy	6	40	BID	14
3	CBR417	6	50	QD	4
4	CBR490	6	75	BID	7
5	CBR490	6	25	BID	7

#### 2.1.4 *L. sigmodontis* jird study II

Eight experimental groups were tested in this study and treatment of the animals started at 16 wpi (Table 2). Group 1 received an equal volume of CBR vehicle for 14 days. Group 2 received 40 mg/kg doxycycline BID for 14 consecutive days (n = 5). Group 3 received 50 mg/kg CBR417 QD for 7 consecutive days (n = 5). Group 4 received 20 mg/kg CBR417 QD for 7 consecutive days (n = 6). Group 5 received 10 mg/kg CBR417 QD for 7 consecutive days (n = 6). Group 6 received 20 mg/kg CBR490 QD for 7 consecutive days (n = 6). Group 7 received 10 mg/kg CBR490 QD for 7 consecutive days (n = 6) and group 8 received BID treatments with 25 mg/kg CBR490 for 7 consecutive days (n = 6). Necropsies were performed at 16 wpt and *L. sigmodontis* worms were isolated and enumerated.

**Table 2:** Dosing regimen for *L. sigmodontis*-infected jirds in study II.

Group	Treatment	n =	Dose (mg/kg)	Daily doses	Duration (d)
1	Vehicle	6	–	BID	14
2	Doxy	5	40	BID	14
3	CBR417	5	50	QD	7
4	CBR417	6	20	QD	7
5	CBR417	6	10	QD	7
6	CBR490	6	20	QD	7
7	CBR490	6	10	QD	7
8	CBR490	6	25	BID	7

#### 2.1.5 Microfilariae counts from peripheral blood

For mf counts, 10 μL of peripheral blood was taken from the saphenous vein in bi-weekly intervals starting at 12 wpi, diluted in 190 μL of red blood cell lysis buffer and stored at room temperature until analysis (BioLegend, San Diego, CA, USA). After resuspension, 10 μL of the suspension was transferred to a microscopic slide and mf were counted using a microscope. If less than 10 mf were counted, the tube was centrifuged at 400 g for 5 min. The supernatant was then discarded, and the pellet was resuspended and completely transferred to a microscopic slide. Mf were counted in the sample using a microscope. Results are presented as number of mf per 10 μL blood.

#### 2.1.6 Necropsies

Necropsies were performed at 16 or 18 wpt. At necropsy, *L. sigmodontis* worms were isolated from the thoracic cavity and quantified as previously described (Hübner et al., 2019b). Briefly, animals were euthanized with an overdose of isoflurane and the peritoneum was opened, followed by a small incision at the diaphragm. Adult worms were collected by flushing the thoracic cavity with PBS. Finally, the pleural cavity was opened completely and inspected for remaining worms. Male and female worms were separated and counted.

#### 2.1.7 Embryograms

Female *L. sigmodontis* adult worms isolated at 16 and 18 wpi were also analyzed for embryogenesis as previously described (Ziewer et al., 2012). Single female adult worms were homogenized in 80 μL PBS and 20 μL Hinkelmann solution (0.5% eosin Y, 0.5% phenol, 0.185% formaldehyde in distilled water) and the developmental stages (eggs, morulae, pretzel, and stretched mf) as well as degenerated embryonic stages were enumerated using a light microscope.

#### 2.1.8 qPCR analysis of *Wolbachia* from *L. sigmodontis* adult female worms

qPCR was performed to validate the depletion of *Wolbachia* bacteria post-treatment using primers for the *Wolbachia* single copy gene *ftsZ* (GenBank Accession No.: AJ010271) and the *L. sigmodontis* actin gene (*act*) (GenBank Accession No.: GU971367) for normalization as previously described (Hübner et al., 2019b). For determination of the *Wolbachia ftsZ*/*act* ratio in adult worms, 10 female worms per animal (if present) were individually frozen at −20 °C for later analysis. DNA from female adult worms was purified using the Qiagen Mini DNA purification kit according to the manufacturer's protocol. The PCR was performed in triplicate by duplex real-time PCR using Qiagen's QuantiNova® on a Rotorgene Q 5-Plex (Qiagen, Hilden, Germany). The following primer pairs (MicroSynth; Switzerland) and TaqMan probes (biomers; Germany) were used: *L. sigmodontis ftsZ* forward 5′-CGATGAGATTATGGAACATATAA-3′, *L. sigmodontis ftsZ* reverse 5′-TTGCAATTACTGGTGCTGC-3′, *L. sigmodontis ftsZ* TaqMan probe 5′6-FAM CAGGGATGGGTGGTGGTACTGGAA-3′TAMRA, *L. sigmodontis act* forward 5′-ATCCAAGCTGTCCTGTCTCT-3′, *L. sigmodontis act* reverse 5′-TGAGAATTGATTTGAGCTAATG-3′, *L. sigmodontis act* TaqMan probe 5′HEX 5′-ACTACCGGTATTGTGCTCGATT-3′TAMRA. The qPCR consisted of 45 cycles with a melting temperature of 95 °C for 5 s and an annealing temperature of 58 °C for 30 s. The standard curve used was a mix of *L. sigmodontis ftsZ* and *act* plasmids.

### 2.2 *Brugia pahangi in vivo* studies

#### 2.2.1 Animal infections

Male jirds (*Meriones unguiculatus*) approximately 6 weeks of age (50–60 g) were purchased from Charles River Laboratories International, Inc., Wilmington, MA USA), and injected intraperitoneally with 200 *B. pahangi* L3 isolated in the Beerntsen laboratory. Animal studies were performed under the University of California, San Francisco Institutional Animal Care and Use Committee (IACUC) approvals AN109629-03 and AN173847-02 and adhered to the guidelines set forth in the NIH Guide for the Care and Use of Laboratory Animals and the USDA Animal Care Policies.

#### 2.2.2 Drug formulations and dosages

CBR417 and CBR490 were provided by Calibr at Scripps Research and dissolved in 40% beta-cyclodextrin at concentrations of 16 mg/mL, 8 mg/mL and 4 mg/mL. Dosing started 23 wpi with 5 groups of jirds: Group 1 (n = 6) was given vehicle QD for 7 days; Group 2 (n = 6) was given 40 mg/kg of CBR417 QD for 7 days; and Groups 3, 4 and 5 were given 40 mg/kg, 20 mg/kg or 10 mg/kg of CBR490, respectively, QD for 7 days (see Table 3).

**Table 3:** Dosing regimen for *B. pahangi-*infected jirds.

Group	Treatment	n =	Dose (mg/kg)	Daily doses	Duration (d)
1	Vehicle	6	–	QD	7
2	CBR417	6	40	QD	7
3	CBR490	6	40	QD	7
4	CBR490	6	20	QD	7
5	CBR490	5	10	QD	7

#### 2.2.3 Pharmacokinetic analyses of jird plasma

For PK sampling during jird/*L. sigmodontis* and *B. pahangi* efficacy experiments, whole blood was collected from the saphenous vein and 8 μL was spotted onto protein saver cards for dried blood spot (DBS) analysis (Whatman 903, Cardiff, UK), and plasma concentration of each compound was determined using LC/MS. Cohorts of *L. sigmodontis*-infected animals were sampled 1, 3, 7, and 24 h post-first and -last morning dose. During jird/*B. pahangi* efficacy experiments, whole blood was collected 3, 24, 48, and 96 h post-first dose; then 0.5 h before the last dose; and 3, 24, 48, and 96 h post-last dose. DBS cards were stored at 4 °C prior to shipment to Accelera (Milano, Italy) for analysis.

#### 2.2.4 Animal necropsies

Animals were necropsied 17 wpt. Adult worms and mf were recovered by opening the body cavity and washing the peritoneal cavity with 100 mL of phosphate buffered saline (PBS). Male and female worms were separated and counted and the number of mf present in the peritoneal cavity was determined by staining with 0.04% methylene blue and counting stained mf on a glass slide using a compound microscope. The mf counts were multiplied by the appropriate dilution factor to calculate the total number of mf from each jird.

#### 2.2.5 Embryograms

To study the effect of treatment on embryogenesis, embryograms were conducted on female worms from each group as previously described (Ford et al., 2009). Each female worm was homogenized in 500 μL of PBS to release the contents of the uterus, followed by counting 10 μL of the extracted embryonic stages using a compound microscope and hemocytometer. At least 200 events were measured for each female worm, and 3–4 females were analyzed per animal from 3–4 animals from each experimental group. The intra-uterine progeny were expressed as the relative proportions of progeny at different stages of development; eggs, developing embryos, pre-microfilariae (pre-mf), mf and degenerated embryonic stages.

#### 2.2.6 Fluorescence *in situ* hybridization (FISH)-based quantification of *Wolbachia* within female worm ovaries

Adult female worms recovered from jird necropsies were frozen on dry ice, thawed and fixed for 20 min at room temperature with 3.2% paraformaldehyde in phosphate buffered saline (pH 7) and 0.1% Tween 20 (PBS-T). *Wolbachia* content in the worm ovaries was quantified as previously described (Serbus et al., 2012; Bakowski et al., 2019). Briefly, ovaries of the worms were dissected out and *Wolbachia* were stained with 0.5 μM of each *Wolbachia*-specific 16S rRNA fluorescence *in situ* hybridization (FISH) probes labeled with Quasar 670 (Biosearch Technologies) complementary to *Wolbachia* 16S rRNA (W2 (Heddi et al., 1999) and Wpan1 (Bakowski et al., 2019), in the presence of unlabeled helper probes (W2H1 and W2H2 (Heddi et al., 1999) and Wpan1H1 and Wpan1H2 (Bakowski et al., 2019)). Stained ovaries were mounted on slides using Vectashield with DAPI mounting medium (Vector Laboratories Inc.). The central plane of each ovary near the distal tip cell was imaged using the Leica SP5 confocal microscope with a 63× objective and *Wolbachia* content was analyzed using Compartmental Analysis in HCS Studio (Thermo Fisher Scientific). Worm germ cell nuclei were identified using DAPI signal and far red (*Wolbachia* 16S rRNA FISH)-stained spots within all identified cells were selected based on their signal intensity above background using the box detection method and fixed thresholding. Average *Wolbachia* spot total intensity per worm cell from each ovary was normalized to averaged data obtained from DMSO treated ovaries to determine % *Wolbachia* elimination (100 ∙ (mean DMSO – sample)/mean DMSO).

#### 2.2.7 Transmission electron microscopy of female *B. pahangi* recovered at necropsy

Female worms recovered from 2–3 jirds per group were fixed in 2.5% glutaraldehyde, 2% paraformaldehyde in 0.1 M sodium cacodylate buffer, pH 7.4 (EMS, USA). Worms were cut into 2–3 mm long pieces in the fixative and incubated for 3 h at room temperature and at 4 °C overnight. Samples were washed thoroughly in the buffer and post-fixed in 1% osmium tetroxide in 0.1 M sodium cacodylate buffer for 1 h. Samples were then washed in the buffer, in distilled water and a series of ethanol dilutions: 30%, 50% (with 5% uranyl acetate), 70%, 95%, for 10 min each, and twice with 100% ethanol for 20 min each. Samples were infiltrated with a gradient of acetone-Embed 812 resin and embedded in 100% resin. After sectioning the solidified blocks, ~70 nm sections were stained with UranyLess (EMS, USA) and lead citrate followed by observation using a FEI Tecnai 12 Spirit transmission electron microscope (Microscopy core facility, NYBC).

### 2.3 qPCR analysis of *Wolbachia* from adult female *B. pahangi*

qPCR was performed to obtain the ratio of *wsp* to *gst* gene copy number, as previously described (Halliday et al., 2014; McGarry et al., 2004). Briefly, individual adult female *B. pahangi* were fixed in RNAlater and stored at 4 °C. For *Wolbachia,* DNA was extracted from worm samples using the DNeasy Blood and Tissue Kit (Qiagen) according to manufacturer's instructions. Levels of *B. pahangi Wolbachia wsp* and *B. pahangi gst* gene copy numbers were quantified using qPCR with *Brugia-*specific primers. The following primers were used to amplify a 164 bp internal sequence of the *Wolbachia wsp* gene (*wsp* primers): *wsp* 420 forward 5′ TGT TGG T(AG)T TGG T(GC)T TGG TG 3^’^; *wsp* 583 reverse 5′ AAC CAA A(AG)T AGC GAG C(CT)C CA 3′. The following primers were used to amplify a 183 bp internal sequence of the *Brugia gst* gene (*gst* primers): *gst* 1377 forward: 5′ TGC TCG CAA ACA TAG TAA TAG T 3′; *gst* 1632 reverse: 5′ATC ACG GAC GCC TTC ACA G 3′.

### 2.4 Statistical analyses

As a primary efficacy parameter, the reduction of *Wolbachia* from *L. sigmodontis* female adult worms was determined. Furthermore, statistical analyses on inhibition of embryogenesis and clearance of mf were performed using GraphPad Prism software Version 8.12 (GraphPad Software, San Diego, USA). Differences between multiple groups that were not normally distributed were tested for statistical significance using Kruskal-Wallis followed by Dunn's multiple comparison test. P-values ≤ 0.05 were considered statistically significant. To determine significance of *Wolbachia* depletion in *B. pahangi* quantified using *Wolbachia*-specific FISH-based staining, a one-way ANOVA with Dunnett's correction for multiple comparisons was used in GraphPad Prism version 8.0.1 (comparison between vehicle and all treatment groups was preselected).

## 3 Results

### 3.1 Short course treatment of *L. sigmodontis*-infected jirds with CBR417 and CBR490 reduces *Wolbachia* levels

Based on the previously described efficacy against *Wolbachia* using short treatment durations with the quinazolines CBR417 and CBR490 in the *L. sigmodontis* mouse model (Bakowski et al., 2019), we performed a more detailed analysis of their efficacy in the *L. sigmodontis* jird model that included parasitological analysis and compared it to doxycycline treatment given for 14 days.

**Fig. 1:**
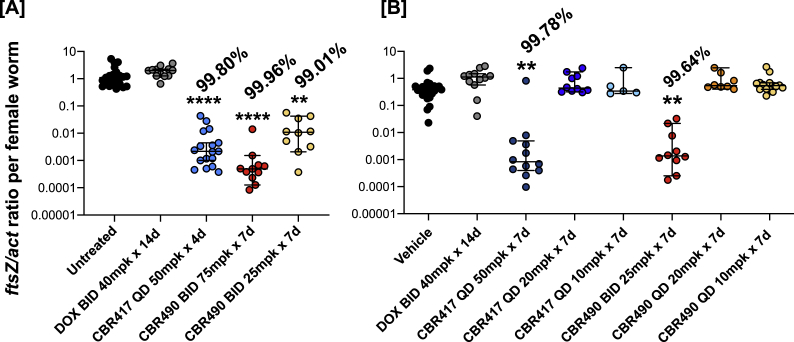
**Short course treatment of *L. sigmodontis*-infected jirds with CBR417 and CBR490 reduces *Wolbachia* levels**. *Wolbachia ftsZ*/filarial actin ratio of female adult worms isolated from jirds infected with *L. sigmodontis* that have been treated with doxycycline (DOX), CBR417, CBR490, vehicle control or left untreated. **A**: 13-week-infected jirds were either left untreated or treated twice a day (BID) with 40 mg/kg (mpk) doxycycline for 14 days, once per day (QD) with 50 mg/kg CBR417 for 4 days or BID with 75 or 25 mg/kg CBR490 for 7 days. **B**: 16-week-infected jirds were either treated BID with vehicle control for 7 days, BID with 40 mg/kg doxycycline for 14 days, QD with 50, 20 or 10 mg/kg CBR417 for 7 days, BID with 25 mg/kg CBR490 for 7 days, or QD with 20 or 10 mg/kg CBR490 for 7 days. Jirds were sacrificed 16 (A) or 18 (B) weeks after treatment. N = 5–6 per group. Analysis for statistical significance was done by Kruskal-Wallis followed by Dunn's multiple comparison post-hoc test. **P < 0.01; ****P < 0.0001.

In line with previous studies testing novel *Wolbachia* targeting compounds, all tested treatment regimens with CBR417 and CBR490 did not lead to any statistically significant decrease in the number of adult worms recovered after 16 or 18 weeks after treatment (Bakowski et al., 2019) (Supplementary Fig. 1). However, 4- and 7-day QD regimens of 50 mg/kg CBR417 (Fig. 1A and B), as well as BID treatments with 75 and 25 mg/kg CBR490 for 7 days (Fig. 1A and B) significantly reduced the *Wolbachia* load in the female worms by more than 99% in comparison to untreated controls. Importantly, these CBR417 and CBR490 treatment regimens were superior to a BID treatment of 40 mg/kg doxycycline given for 14 days, which resulted in no *Wolbachia* reduction at 16 and 18 wpt in comparison to untreated controls (Fig. 1A and B). Lower QD doses of 20 and 10 mg/kg CBR417 or CBR490 given for 7 days did not deplete *Wolbachia* in the female worms and was therefore not superior over doxycycline treatment (Fig. 1A and B). These data indicate that QD administrations of 50 mg/kg CBR417 as short as 4 days and BID administrations of 75 and 25 mg/kg CBR490 for 7 days are highly efficacious in depleting *Wolbachia* endosymbionts.

### 3.2 Embryogenesis is inhibited by CBR417 and CBR490 treatment in the *L. sigmodontis* jird model

**Fig. 2:**
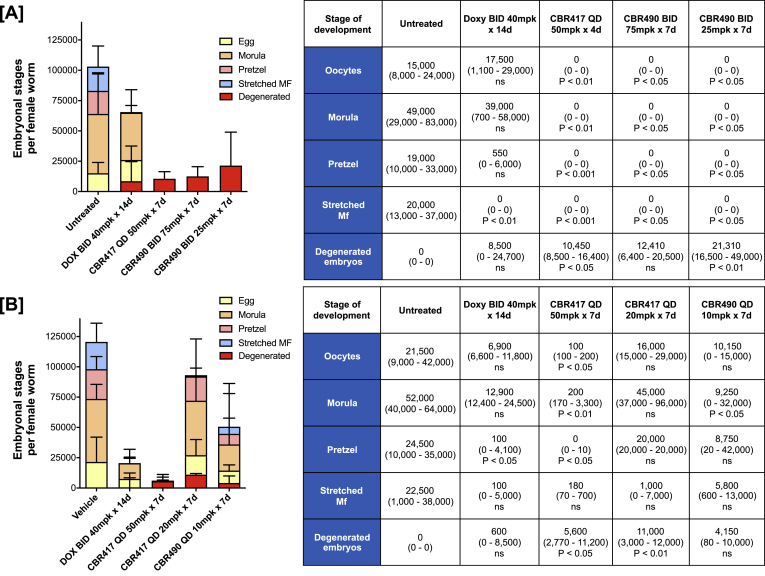
**Embryogenesis is inhibited by CBR417 and CBR490 treatment in the *L. sigmodontis*jird model.** Embryonic stages (oocyte, morula, pretzel, stretched mf and degenerated embryos) per female adult worms isolated from jirds infected with *L. sigmodontis* that were treated with doxycycline (DOX), CBR417, CBR490, vehicle control or left untreated. **A**: 13-week-infected jirds were either left untreated or treated bi-daily (BID) with 40 mg/kg doxycycline for 14 days, once per day (QD) with 50 mg/kg CBR417 for 4 days or BID with 75 or 25 mg/kg CBR490 for 7 days. **B**: 16-week-infected jirds were either treated BID with vehicle control for 7 days, BID with 40 mg/kg doxycycline for 14 days, QD with 50 or 20 mg/kg CBR417 for 7 days, or QD with 10 mg/kg CBR490 for 7 days. Jirds were sacrificed 16 (A) or 18 (B) weeks after treatment. Analysis for statistical significance was done by Kruskal-Wallis followed by Dunn's multiple comparison post-hoc test.

Since *Wolbachia* bacteria are essential for filarial fecundity, embryogenesis was analyzed in female adult worms isolated from jirds infected with *L. sigmodontis* that were treated with different regimens of CBR417 and CBR490 and compared to untreated and doxycycline-treated animals (Hoerauf et al., 2000). Embryogenesis within the uteri of female worms from animals treated with 50 mg/kg of CBR417 QD for 4 and 7 days or with CBR490 BID at 75 or 25 mg/kg for 7 days significantly inhibited embryogenesis and resulted mainly in degenerated embryos (Fig. 2A and B). Lower QD doses of CBR417 (20 mg/kg, 7 days) and CBR490 (10 mg/kg, 7 days) had no significant impact on oocyte, pretzel, and stretched mf counts (Fig. 2B), but resulted in a significant increase in degenerated embryos in CBR417 (20 mg/kg QD for 7 days)-treated animals and a significant reduction in the morula stages of CBR490 (10 mg/kg QD for 7 days)-treated animals compared to vehicle controls. Doxycycline treatment reduced the total number of embryonic stages in comparison to vehicle/untreated controls, leading to statistically significant changes in one of the two experiments in regard to the number of stretched mf in the uteri (Fig. 2A) and pretzel stages (Fig. 2B). These data indicate that treatments of 4 and 7 days with CBR417 or CBR490, respectively, can lead to a complete inhibition of the embryogenesis in the *L. sigmodontis* jird model.

### 3.3 Microfilariae levels decline in response to CBR417 and CBR490 treatment

**Fig. 3:**
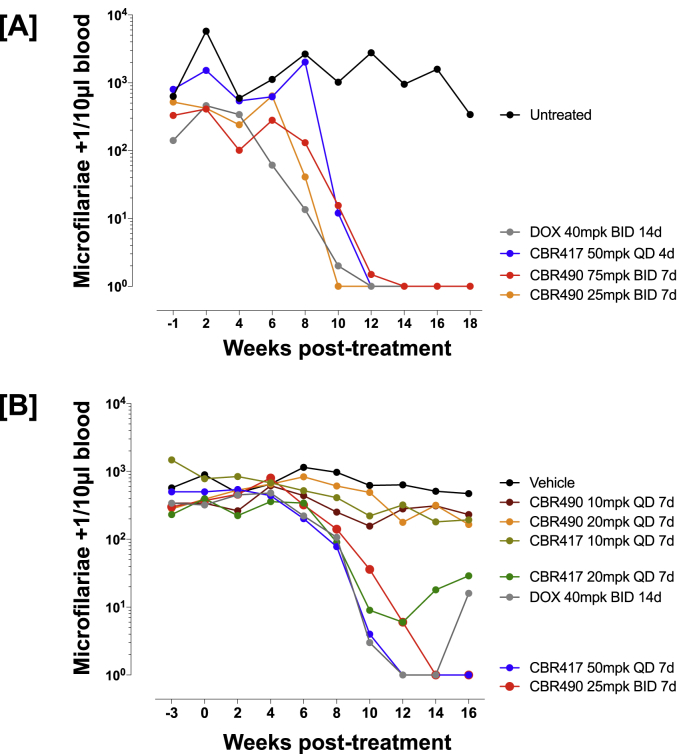
**Microfilariae levels decline in response to CBR417 and CBR490 treatment.** Mf count per 10 μL of blood drawn from jirds infected with *L. sigmodontis* and that have been treated with doxycycline (DOX), CBR417, CBR490, vehicle control or left untreated. **A**: 13-week-infected jirds were either left untreated () or treated bi-daily (BID) with 40 mg/kg doxycycline for 14 days (), once per day (QD) with 50 mg/kg CBR417 for 4 days () or BID with 75 () or 25 mg/kg () CBR490 for 7 days. **B**: 16-week-infected jirds were either treated BID with vehicle control for 7 days (), BID with 40 mg/kg doxycycline for 14 days (), QD with 50 (), 20 () or 10 () mg/kg CBR417 for 7 days, QD with 20 () or 10 () mg/kg CBR490 for 7 days or BID with 25 mg/kg CBR490 for 7 days (). Jirds were sacrificed 16 (A) or 18 (B) weeks after treatment. N = 5–6 per group.

Since embryogenesis was inhibited in female adult *L. sigmodontis* worms after 4–7 days of CBR417 and CBR490 treatment, the kinetics of peripheral blood mf clearance were also investigated. Starting 8–10 weeks after treatment, peripheral blood mf numbers began to decline in animals that received CBR417 at 50 mg/kg QD for 4 or 7 days, as well as 20 mg/kg for 7 days (Fig. 3A and B). Similarly, treatment with CBR490 given BID at 75 mg/kg and 25 mg/kg also started to reduce the mf levels around 8–10 wpt. Notably, CBR417 given at 50 mg/kg QD for 4 or 7 days and CBR490 given BID at 75 or 25 mg/kg completely cleared the peripheral blood mf by 16–18 wpt, while treatment with 40 mg/kg doxycycline given BID for 14 days initially cleared mf in 9 out of 11 animals by 12 wpt, but then led to a rebound of the mf with 5 out of 11 animals being mf positive by 16–18 wpt (Fig. 3A and B). Lower QD doses of CBR490 and CBR417 of 10–20 mg/kg did not significantly reduce the mf load in comparison to the vehicle controls (Fig. 3B). Importantly, the slow decline in the mf burden of CBR417 and CBR490-treated animals in addition to the shown inhibition of the embryogenesis indicate that both candidates lack a strong direct acting microfilaricidal activity; this is important from the point of view of reducing risk of potential serious adverse events.

### 3.4 Short course treatment of *B. pahangi*-infected jirds reduces microfilarial shedding

**Fig. 4:**
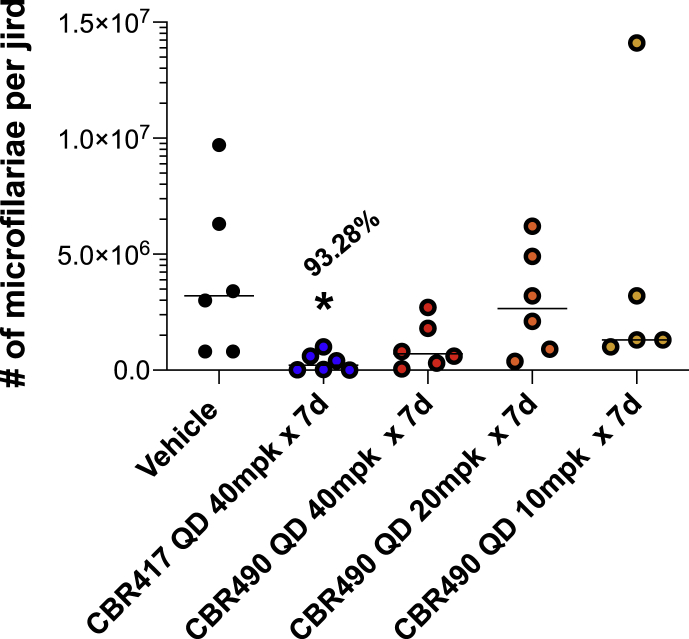
***B. pahangi* microfilariae recovered at necropsy** a**re significantly reduced in jirds treated with 40 mg/kg CBR417 given QD for 7 days (P < 0.05).** Mf were recovered from the peritoneal cavities and counted from each jird 17 wpt.

In parallel with the *L. sigmodontis* jird model, drug efficacy was analyzed in jirds infected with *B. pahangi*. Similar to the results observed for the *L. sigmodontis*-infected jirds, adult worm recovery from *B. pahangi*-infected jirds treated with CBR417 nor CBR490 was not affected at the time of necropsy (17 wpt). However, the number of mf from the peritoneal cavities of *B. pahangi*-infected jirds was significantly lower (P < 0.05) in animals treated with 40 mg/kg CBR417 QD for 7 days but not with the other treatment groups (Fig. 4). After female worms were recovered from the peritoneal cavities, each worm was incubated individually overnight at 37 °C with 5% CO_2_ and the number of mf released by each female worm was counted. Consistent with the reduced number of mf recovered at necropsy, 89% of the females from animals treated with CBR417 did not shed mf overnight (Table 4).

**Table 4:** CBR417 reduced the number of *B. pahangi* microfilariae that were released from female worms recovered from jirds at necropsy. Female worms were recovered from jirds and cultured overnight to assess the number of mf released by each worm. 89% of female worms from animals treated with CBR417 did not shed mf (8 of 9 female worms).

Treatment	# females shedding mf	# females not shedding mf	% females not shedding mf
Vehicle	7	9	56%
CBR417 QD 40 mpk x 7d	1	8	89%
CBR490 QD 40 mpk x 7d	6	6	50%
CBR490 QD 20 mpk x 7d	13	4	24%
CBR490 QD 10 mpk x 7d	8	4	33%

### 3.5 Development of microfilariae are significantly reduced in animals treated with both CBR417 and CBR490

**Fig. 5:**
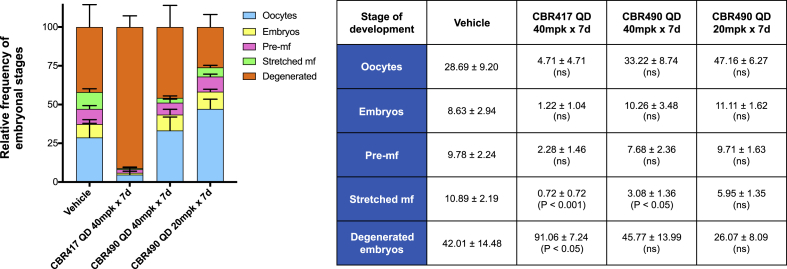
**Embryograms of female*****B. pahangi*****show that CBR417 and CBR490 affect late stages of developing mf.**Both CBR417 and CBR490 (40 mg/kg QD for 7 days) significantly reduced the number of later-staged mf. Female worms recovered from jirds treated with CBR417 also had significantly more degenerated embryos in their ovaries. Embryonic stages (oocytes, embryos, pre-mf, stretched mf and degenerated embryos) per female adult worm were removed from jirds infected with *B. pahangi* and counted from the ovaries and uteri of individual worms. Results are shown as mean relative frequencies of embryonic stages ± SEM

Embryograms revealed that the number of stretched mf were significantly reduced in animals treated with CBR417 (P < 0.001) and CBR490 (P < 0.01) when both groups were dosed at the same dosage of 40 mg/kg QD for 7 days. CBR417 also caused an increase in the number of deformed embryos (P < 0.01) (Fig. 5).

### 3.6 Transmission electron micrographs reveal damage to *B. pahangi* microfilariae developing within the treated female ovaries

**Fig. 6:**
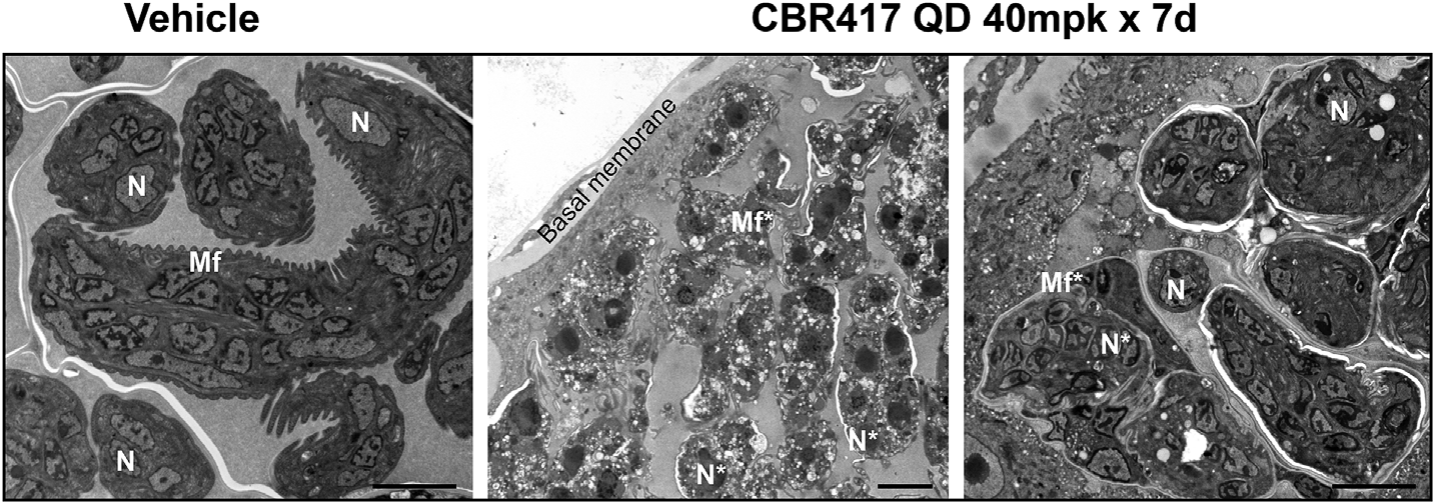
**Transmission electron micrographs reveal extensive damage to the developing *B. pahang*i microfilariae in ovaries of worms from jirds treated with CBR417 QD 40 mg/kg for 7 days.** N = nucleus; N* = abnormal nucleus; Mf = microfilariae developing in uteri; Mf* = abnormal microfilariae in uteri; bar = 4 μm.

Consistent with the results of the embryograms and reduced number of mf that were shed from female *B. pahangi* worms that were recovered at necropsy, ultrastructural analysis revealed extensive damage in ovaries of female worms from jirds treated with CBR417 (40 mg/kg QD for 7 days). Numerous vacuoles, large empty tissue spaces and irregular membranous structures were observed in embryos from female worms recovered from these treated animals compared to the ultrastructural organization seen in worms from the vehicle group (Fig. 6).

### 3.7 Fluorescence analysis reveals significant reduction of *Wolbachia* in *B. pahangi* female worms from jirds treated with CBR417 and CBR490

**Fig. 7:**
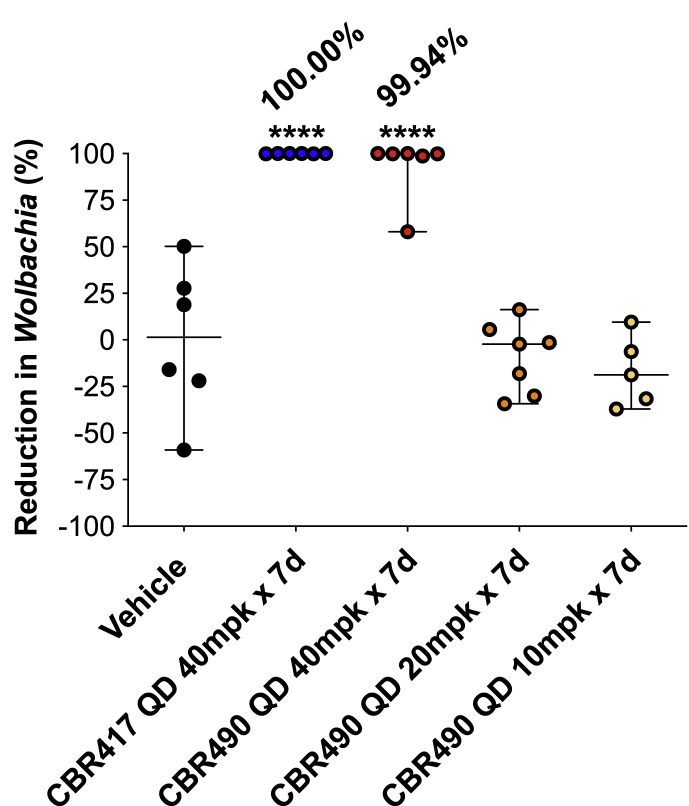
**High content imaging of ovaries showa highly significant reduction in*****Wolbachia*****from female*****B. pahangi*****worms recovered from jirds treated with 40 mg/kg QD x 7 days CBR417 (P < 0.0001) and CBR490 (P < 0.0001)**. Ovaries from individual females were removed and stained with *Wolbachia* specific 16S rRNA probes using fluorescence *in situ* hybridization (FISH) and DAPI. Ovaries were imaged using confocal microscopy and *Wolbachia* quantified using high content image analysis. Means ± SD (average signal from 1 to 2 ovaries from 5 to 7 worms per treatment) are shown, and mean elimination (%) is reported. To assess significance between the vehicle control and treatment groups a one-way ANOVA with Dunnett's multiple comparisons test was used (****P < 0.0001).

Ovaries were assessed for *Wolbachia* titers from individual female worms recovered from jirds at necropsy (17 wpt) using *Wolbachia*-specific 16S rRNA FISH staining and high content image analysis. Female worms recovered from animals treated with 40 mg/kg QD x 7 days of CBR417 or CBR490 had significantly lower *Wolbachia* titers (>99.99% and 92.77% mean *Wolbachia* elimination, respectively; P < 0.0001) compared to worms from vehicle treated animals (Fig. 7).

### 3.8 CBR417 significantly reduces *Wolbachia* titers in adult female and male *B. pahangi* filariae

**Fig. 8:**
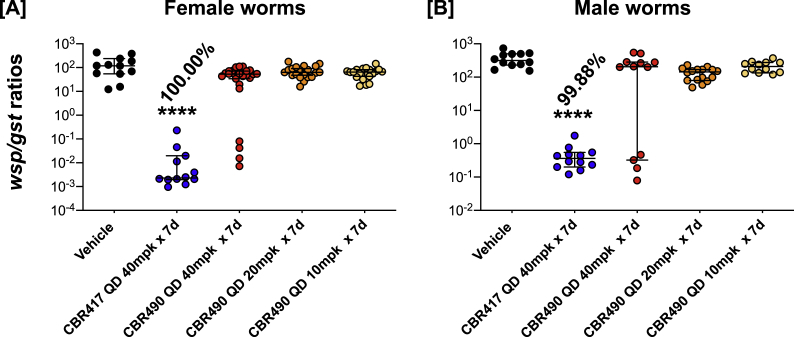
**CBR417is highly effective in reducing*****Wolbachia*****titers in adult*****B. pahangi*****.** qPCR analysis of individual worms showed there was a >99% reduction in titers in female (A) and male (B) worms compared to worms from vehicle-treated animals (****P < 0.0001).

CBR417 given to *B. pahangi*-infected jirds at doses of 40 mg/kg QD for 7 days had a significant effect on *Wolbachia* titers in both male and female worms (Fig. 7A and B). qPCR analysis of individual worms showed there was a >99% reduction in titers in female and male worms compared to worms from vehicle-treated animals (Fig. 8A and B).

### 3.9 CBR417 and CBR490 exposures achieved in jirds during *in vivo* experiments

Sparse PK sampling during *in vivo* efficacy experiments carried out in jirds infected with either *L. sigmodontis* (Supplement 2A) or *B. pahangi* (Supplement 2B) confirmed relative exposures of CBR417 and CBR490 achieved during these studies. Exposures of both compounds matched previously-published PK profiles observed in mice (Bakowski et al., 2019) and a slight accumulation of CBR417 was observed after both 4 (Supplement 2A) and 7 (Supplement 2B) days of QD dosing. Notably, lower doses of CBR490 in *B. pahangi*-infected jirds (20 or 10 mg/kg 7 days QD) (Supplement 2B) failed to reach exposures markedly above anti-*B. pahangi Wolbachia* EC50 previously determined *ex vivo* (Bakowski et al., 2019).

## 4 Discussion

Our study demonstrates that the quinazoline drug candidates CBR417 and CBR490 are highly effective in two rodent models of filariasis. In agreement with a recently published study (Bakowski et al., 2019), short term regimens of 7 days or less with CBR417 and CBR490 achieved significant *Wolbachia* depletions of >99%. While the previous study by Bakowski et al. focused on the depletion of the *Wolbachia* endosymbionts ~4 weeks after treatment, the present study used two models of chronic filariasis, which allowed a long-term analysis of the impact of the CBR417 and CBR490 treatment regimens on *Wolbachia* depletion as well as adult worm burden, microfilaremia and embryogenesis.

Limitations of our study are due to the usage of surrogate filarial species in animal models for the preclinical analysis of drugs intended for human filariasis, with filarial species that may possess a different susceptibility for the drug candidates, altered PK/PD relations in the rodent hosts and differences in the anatomical locations where the adult filariae are found. Nevertheless, *L. sigmodontis* and *Brugia* rodent models have been used in a range of preclinical studies, including a human progression study with the preclinical candidate ABBV-4083 (DNDi, 2019; Fischer et al., 2019; Hong et al., 2019; Hübner et al., 2019a; Jacobs et al., 2019; Taylor et al., 2019). Doxycycline, the first proven safe macrofilaricidal treatment for human filariasis was also tested in the *L. sigmodontis* model and was comparable to what was found in human studies (Hoerauf et al., 1999), i.e. clearance of *Wolbachia*, microfilaremia and inhibition of embryogenesis, indicating that sufficient drug exposures can be achieved in subcutaneous nodules of onchocerciasis or lymph vessels in the case of lymphatic filariasis. Given the suboptimal doxycycline treatment regimen of only 2 weeks in our present study, we did not observe a clearance of *Wolbachia* within the female adult worms at 16–18 wpt. A previous study from our group showed that this doxycycline regimen leads to an initial reduction of *Wolbachia*, which is not maintained and leads to a rebound of *Wolbachia* (Hübner et al., 2019b). Accordingly, this doxycycline regimen also did not completely clear the peripheral mf.

In line with previous studies by our groups and others (Hong et al., 2019; Jacobs et al., 2019; Taylor et al., 2019), no differences were observed in the adult worm burden following all tested anti-*Wolbachia* treatment regimens (Bakowski et al., 2019; Taylor et al., 2019), including those with CBR490 and CBR417. Macrofilaricidal efficacy of *Wolbachia*-targeting compounds is slow acting and occurs in humans harboring filarial infection only after ~2 years (Hoerauf et al., 2009, 2011; Debrah et al., 2015). Nevertheless, results from the present study indicate that in line with the successful depletion of *Wolbachia* in the adult filarial worms, a gradual decline of peripheral microfilaremia over a period of 16–18 weeks was observed that was accompanied by the complete inhibition of embryogenesis. Such a slow removal of peripheral blood mf is beneficial, as it may prevent drug-induced serious adverse events that are associated with a fast clearance of the mf, for example, as they occur following DEC treatment in onchocerciasis patients. Furthermore, *Wolbachia*-targeting drugs should not impact *L. loa*, as this filarial species lacks the endosymbiotic bacteria (Desjardins et al., 2013), preventing the risk of serious adverse events in areas co-endemic for loiasis. Although we did not confirm that CBR490 and CBR417 treatments deplete *Wolbachia* from male *L. sigmodontis* worms, our experiments with *B. pahangi* indicate that both compounds are effective in male as well as female filariae. Nevertheless, pathology in onchocerciasis is driven by the dying mf and not the adult filariae, indicating that sterilization of the female adult worms and lack of mf release should prevent the occurrence or progression of pathology.

Comparison of the different regimens used for CBR490 and CBR417 in our study further showed that CBR417 given QD at 50 mg/kg for as short as 4 days and CBR490 given BID at doses as low as 25 mg/kg for 7 days mediated the clearance of >99% of *Wolbachia*, leading to amicrofilaremia and complete inhibition of embryogenesis in all animals treated. In contrast, QD treatments with CBR490 or lower doses of 10–20 mg/kg CBR417 were not efficacious in line with inadequate PK exposure achieved at those lower doses in relation to EC90s against *B. pahangi Wolbachia* previously determined in *ex vivo* experiments (Bakowski et al., 2019). Moreover, our study demonstrated the importance of longer observation periods of at least 4 months after treatment; this was illustrated in the suboptimal 2-week treatment with doxycycline and the low dosages of CBR417, which led to an initial drop in peripheral blood mf levels (Fig. 3) and a reduction of embryonic stages (Fig. 2), and a later rebound of mf levels (Fig. 3).

This was similarly shown in a previous study where the anti-*Wolbachia* drug candidate ABBV-4083 was tested in the *L. sigmodontis* model (Hübner et al., 2019b). Mouse models used in the earlier preclinical studies were predictive of the initial *Wolbachia*-depleting effect, but were limited in analysis of the long-term effects, which is essential to observe the possible rebound of *Wolbachia* and mf. Therefore, the impact of anti-wolbachial compound treatment during a patent, chronic infection and on filarial embryogenesis as well as kinetics of mf clearance can only be analyzed in jirds, as they have an increased susceptibility for infection with *L. sigmodontis* as well as *B. pahangi* in comparison to wildtype mice (Morris et al., 2013). Thus, the two jird models of filarial infection used in the current study are a valuable, advanced model system to assess the longer-term impact that *Wolbachia* have on microfilarial output and disruption of female fecundity.

In summary, our study demonstrates the value of the two jird models that allow chronic, patent filarial infections to assess anti-wolbachial compounds and their impact on long-term *Wolbachia* and mf clearance as well as embryogenesis. Especially in regard to the potential rebound of *Wolbachia* and mf using suboptimal treatment regimens, such models are essential. Using these models, we demonstrate that CBR417 and CBR490 are two promising novel drug candidates that deplete *Wolbachia* endosymbionts of filarial nematodes and clear circulating mf via the disruption of embryogenesis. Thus, CBR417 and CBR490 are candidates for the treatment of human filariasis that allow treatment regimens of 7 days or less.

## 5 Authors’ contributions

**Conceptualization:** MPH, CWM, AH, SL, JAS.

**Formal analysis:** MPH, EG, CAB, EG, MK, DV, AS, HMP, WS, JDT, JAS.

**Methodology:** MPH, EG, IV, CAB, KCL, MK, AE, SJF, MF, NT, DV, AS, VC, MAB, AKW, HMP, BB, WS, LC, JDT, JAS.

**Project administration:** MPH, CWM, BB, WS, MJT, JDT, AH, SL, JAS.

**Resources:** MPH, CWM, BB, WS, MJT, JDT, AH, SL, JAS.

**Supervision:** MPH, CWM, BB, WS, MJT, JDT, AH, SL, JAS.

**Writing of original draft:** MPH, AE, SJF, JAS.

**Writing - review & editing:** MPH, CAB, MAB, CWM, JDT, BB, WS, MJT, AH, SL, JAS.

## Appendix A Supplementary data

Suppl. Fig. 1 **Short course treatment of *L. sigmodontis*-infected jirds with CBR417 and CBR490 has no significant impact on adult worm recovery**. Adult worm burden from jirds infected with *L. sigmodontis* that have been treated with doxycycline (DOX), CBR417, CBR490, vehicle control or left untreated. **(A)** 13-week-infected jirds were either left untreated or treated twice a day (BID) with 40 mg/kg doxycycline for 14 days, once per day (QD) with 50 mg/kg CBR417 for 4 days or BID with 75 or 25 mg/kg CBR490 for 7 days. **(B)** 16-week-infected jirds were either treated BID with vehicle control for 7 days, BID with 40 mg/kg doxycycline for 14 days, QD with 50, 20 or 10 mg/kg CBR417 for 7 days, BID with 25 mg/kg CBR490 for 7 days, or QD with 20 or 10 mg/kg CBR490 for 7 days. Jirds were sacrificed 16 (A) or 18 (B) weeks after treatment. N = 5–6 per group. Analysis for statistical significance was done by Kruskal-Wallis followed by Dunn's multiple comparison post-hoc test. P < 0.05. Suppl. Fig. 2. Sparse pharmacokinetic (PK) sampling during in vivo studies confirms relative exposures of CBR417 and CBR490. *L. sigmodontis*-infected (A) and *B. pahangi*-infected (B) jirds were dosed orally with CBR417 or CBR490 as indicated. Cohorts of animals were sampled at designated intervals post first (teal lines) and last morning dose (orange lines). Means ± SD (n = 2–3 animals per timepoint) are shown. For reference, EC90s of CBR417 and CBR490 previously determined against *B. pahangi Wolbachia* in *ex vivo* experiments are shown with dashed red lines. Dashed orange lines indicate exposures falling beneath the threshold of detection. Suppl. Fig. 1
