# Supplementary figures and images for: Short-course quinazoline drug treatments are effective in the Litomosoides sigmodontis and Brugia pahangi jird models
Source: Int J Parasitol Drugs Drug Resist. 2019 Dec 10;12:18–27. doi: 10.1016/j.ijpddr.2019.12.001 (PMC6931063; doi:10.1016/j.ijpddr.2019.12.001)

Supplementary Figure 1

[A]

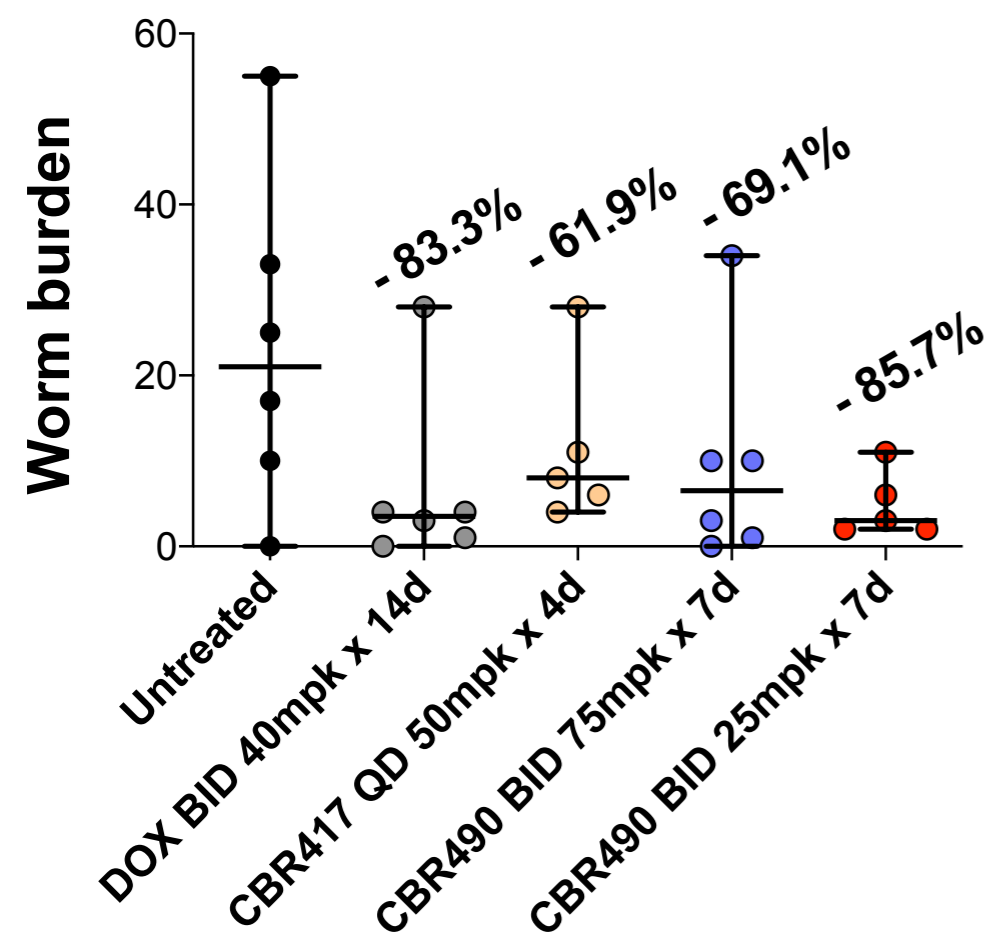

[B]

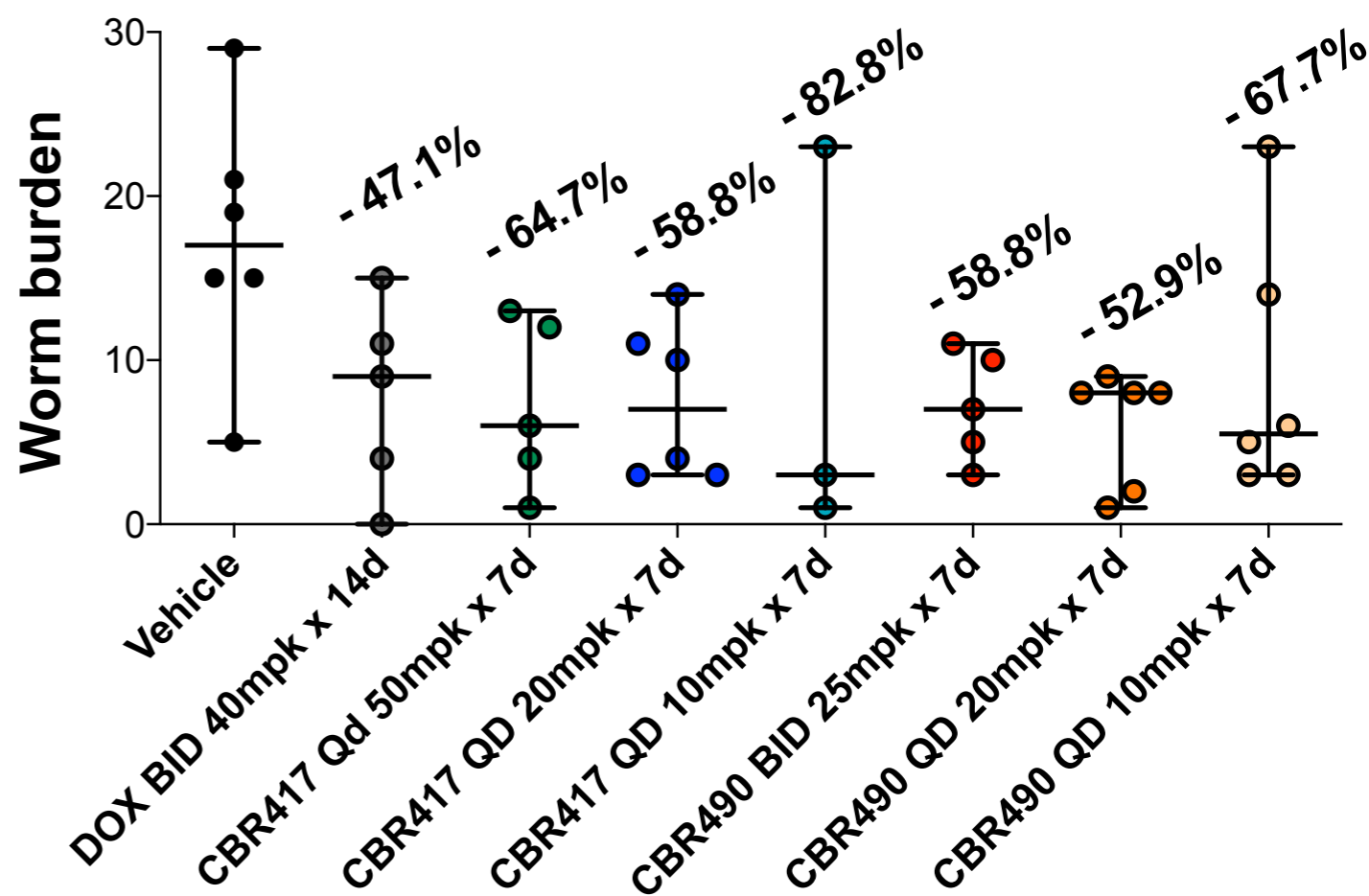

# Supplementary Figure 2

[A]

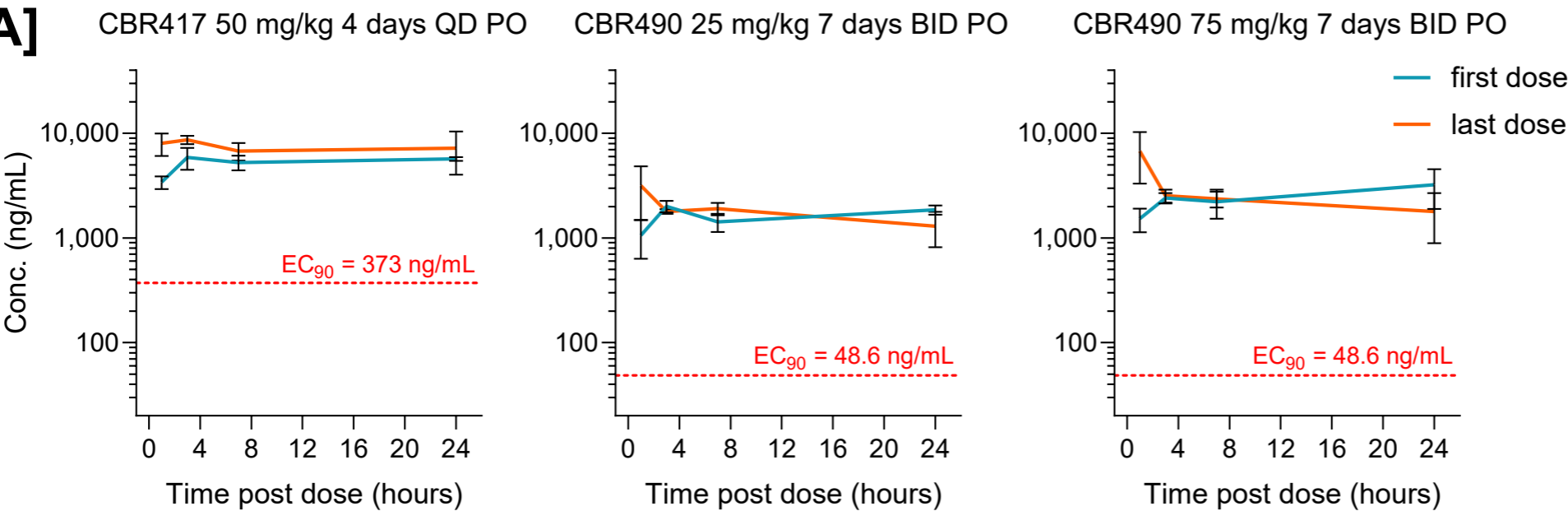

[B]

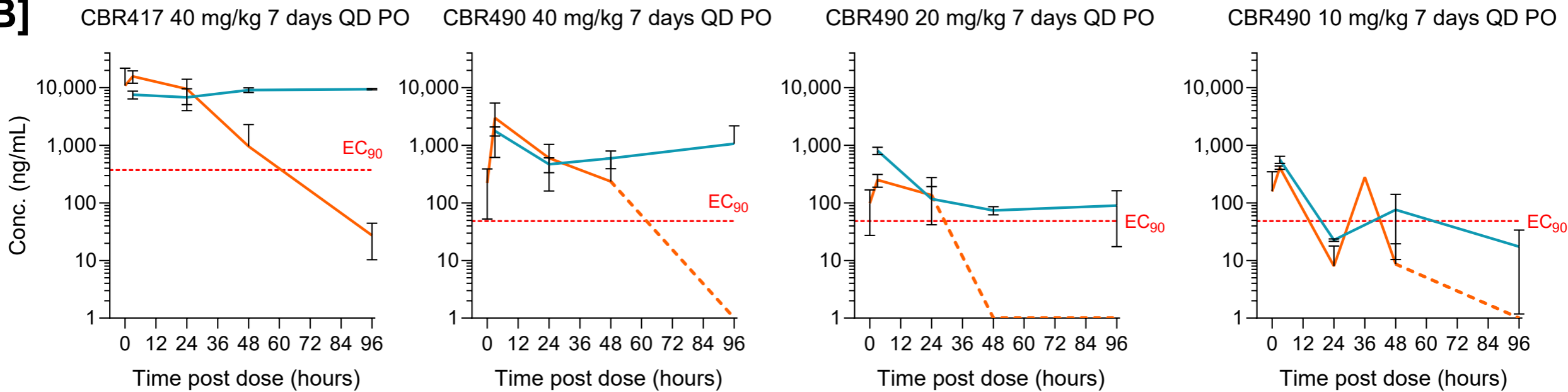

Supplement: Suppl. Fig. 1 — Short course treatment of L. sigmodontis-infected jirds with CBR417 and CBR490 has no significant impact on adult worm recovery. Adult worm burden from jirds infected with L. sigmodontis that have been treated with doxycycline (DOX), CBR417, CBR490, vehicle control or left untreated. (A) 13-week-infected jirds were either left untreated or treated twice a day (BID) with 40 mg/kg doxycycline for 14 days, once per day (QD) with 50 mg/kg CBR417 for 4 days or BID with 75 or 25 mg/kg CBR490 for 7 days. (B) 16-week-infected jirds were either treated BID with vehicle control for 7 days, BID with 40 mg/kg doxycycline for 14 days, QD with 50, 20 or 10 mg/kg CBR417 for 7 days, BID with 25 mg/kg CBR490 for 7 days, or QD with 20 or 10 mg/kg CBR490 for 7 days. Jirds were sacrificed 16 (A) or 18 (B) weeks after treatment. N = 5–6 per group. Analysis for statistical significance was done by Kruskal-Wallis followed by Dunn's multiple comparison post-hoc test. P < 0.05. Suppl. Fig. 2. Sparse pharmacokinetic (PK) sampling during in vivo studies confirms relative exposures of CBR417 and CBR490. L. sigmodontis-infected (A) and B. pahangi-infected (B) jirds were dosed orally with CBR417 or CBR490 as indicated. Cohorts of animals were sampled at designated intervals post first (teal lines) and last morning dose (orange lines). Means ± SD (n = 2–3 animals per timepoint) are shown. For reference, EC90s of CBR417 and CBR490 previously determined against B. pahangi Wolbachia in ex vivo experiments are shown with dashed red lines. Dashed orange lines indicate exposures falling beneath the threshold of detection. [file mmc1.pdf]
